# NAD(P)H:Quinone Oxidoreductase 1 (NQO1) Localizes to the Mitotic Spindle in Human Cells

**DOI:** 10.1371/journal.pone.0044861

**Published:** 2012-09-11

**Authors:** David Siegel, Jadwiga K. Kepa, David Ross

**Affiliations:** Department of Pharmaceutical Sciences, Skaggs School of Pharmacy and Pharmaceutical Sciences, University of Colorado Anschutz Medical Campus, Aurora, Colorado, United States of America; University of Iowa, United States of America

## Abstract

NAD(P)H:quinone oxidoreductase 1 (NQO1) is an FAD containing quinone reductase that catalyzes the 2-electron reduction of a broad range of quinones. The 2-electron reduction of quinones to hydroquinones by NQO1 is believed to be a detoxification process since this reaction bypasses the formation of the highly reactive semiquinone. NQO1 is expressed at high levels in normal epithelium, endothelium and adipocytes as well as in many human solid tumors. In addition to its function as a quinone reductase NQO1 has been shown to reduce superoxide and regulate the 20 S proteasomal degradation of proteins including p53. Biochemical studies have indicated that NQO1 is primarily located in the cytosol, however, lower levels of NQO1 have also been found in the nucleus. In these studies we demonstrate using immunocytochemistry and confocal imaging that NQO1 was found associated with mitotic spindles in cells undergoing division. The association of NQO1 with the mitotic spindles was observed in many different human cell lines including nontransformed cells (astrocytes, HUVEC) immortalized cell lines (HBMEC, 16HBE) and cancer (pancreatic adenocarcinoma, BXPC3). Confocal analysis of double-labeling experiments demonstrated co-localization of NQO1with alpha-tubulin in mitotic spindles. In studies with BxPc-3 human pancreatic cancer cells the association of NQO1 with mitotic spindles appeared to be unchanged in the presence of NQO1 inhibitors ES936 or dicoumarol suggesting that NQO1 can associate with the mitotic spindle and still retain catalytic activity. Analysis of archival human squamous lung carcinoma tissue immunostained for NQO1 demonstrated positive staining for NQO1 in the spindles of mitotic cells. The purpose of this study is to demonstrate for the first time the association of the quinone reductase NQO1 with the mitotic spindle in human cells.

## Introduction

NAD(P)H:quinone oxidoreductase 1 (NQO1, DT-diaphorase, EC 1.6.99.2) is a homodimeric flavoprotein that utilizes either NADH or NADPH and catalyzes the 2-electron reduction of a broad range of substrates most notably quinones [1]. The two-electron reduction of quinones to hydroquinones by NQO1 is believed to be primarily a detoxification reaction since it bypasses the formation of the highly reactive semiquinone [1]. In many cases, however, the reduction of quinones by NQO1 results in the formation cytotoxic hydroquinones and the bioactivation of quinone prodrugs by NQO1 has been utilized as a strategy to target NQO1-rich cancer cells [2].

In normal tissues, NQO1 is expressed at relatively high levels in epithelial tissues, vascular endothelium and adipocytes while in cancer, NQO1 is expressed at high levels in many solid tumors including lung (NSCLC), breast and pancreatic [3], [4]. In humans, the NQO1*2 polymorphism plays a major role in governing basal protein levels of NQO1 [5]. The NQO1*2 polymorphism results in a proline to serine amino acid substitution at position 187 in NQO1 and this mutant protein undergoes rapid polyubiquitination by the E3 ubiquitin ligase STUB1/CHIP with subsequent proteasomal degradation [6], [7]. Individuals homozygous for the NQO1*2 polymorphism are NQO1 null, while intermediate levels of NQO1 protein are found in individuals with the heterozygous genotype [5]. NQO1 is under transcriptional regulation by the Keap1/NRF2 pathway and upregulation of NQO1 mRNA or protein has been used extensively as a biomarker for NRF2 activation [8], [9]. Upregulation of NQO1 may protect the cell from oxidative damage due to the ability of NQO1 to reduce superoxide to hydrogen peroxide and generate antioxidant forms of vitamin E and co-enzyme Q [10], [11], [12].

In addition to its role as an antioxidant enzyme, NQO1 has been shown to protect a wide range of proteins including p53 from ubiquitin-independent 20 S proteasomal degradation [13], [14]. The protection of target proteins by NQO1 from 20 S proteasomal degradation is dependent upon the redox state of NQO1 since treatment with the NQO1 inhibitor dicoumarol has been shown to enhance the 20 S proteasomal degradation of several target proteins [13], [14].

NQO1 is predominately located in the cytoplasm but low levels of NQO1 have been found in the nucleus under normal conditions [15]. Under conditions of stress NQO1 has been shown to migrate to the nucleus where it is hypothesized that NQO1 may protect p53 against 20 S proteasomal degradation [16]. In experiments designed to monitor the subcellular distribution of NQO1 in human cells by confocal microscopy we discovered that NQO1 could also be found in association with the mitotic spindle.

## Results

**Figure 1:**
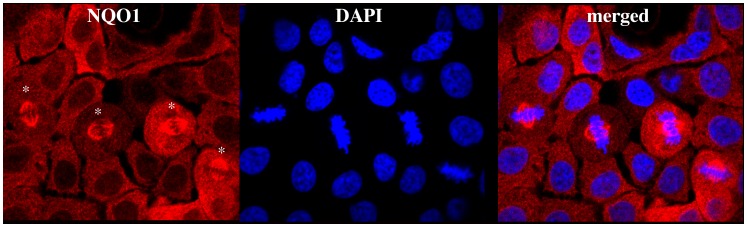
Immunofluorescent staining of NQO1 in BxPc-3 human pancreatic cancer cells. Immunostaining for NQO1 reveals cytosolic localization and intense staining of mitotic spindles in cells undergoing division (*).

Immunofluorescent staining of the human pancreatic adenocarcinoma cell line BxPc-3 for NQO1 revealed that NQO1 is located primarily in the cytosol of these cells (Fig. 1). However, in BxPc-3 cells undergoing mitosis intense immunostaining for NQO1 was observed on the mitotic spindles (Fig. 1). Our source of anti-NQO1 monoclonal antibody for these studies was conditioned tissue culture supernatant from mouse hybridoma clone A180. In control studies, we utilized unconditioned media (RPMI1640 containing 10% fetal bovine serum) in place of hybridoma clone A180 supernatant and in these studies no immunofluorescent staining of NQO1in BxPc3 cells or spindles could be observed (Fig. S1). In addition, no observable difference in immunostaining of NQO1 including spindles could be detected in BxPc-3 cells between tissue culture supernatant from hybridoma clone A180 or commercially prepared affinity purified mouse IgG from hybridoma clone A180 (Fig. S2).

**Figure 2:**
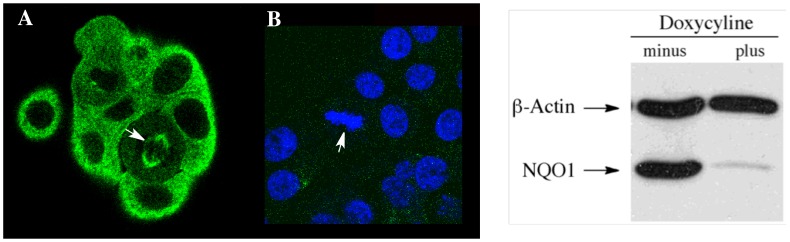
Knockdown of NQO1 eliminates immunostaining of NQO1 in BxPc-3 cells. Knockdown of NQO1 in BxPc-3 cells was performed using a doxycyline- inducible anti-NQO1 shRNA methodologies. BxPc-3 cells were immunostained for NQO1 (green) in the absence (panel A) and presence (panel B) of doxycyline pretreatment. DAPI staining was included in panel B to aid in the location of cells. Right panel: immunoblot demonstrating near complete knockdown of NQO1 protein in the presence of doxycycline. Arrows indicate mitotic cells.

To confirm that NQO1 was the target of monoclonal antibody A180 we generated a BxPc-3 cell line stably expressing a doxycycline-inducible anti-NQO1shRNA expression vector. The growth of this genetically modified BxPc3 cell line in doxycyline for 7 days resulted in near complete knockdown of NQO1 protein expression as confirmed by immunoblotting for NQO1 (Fig 2). As expected, doxycycline treated cells showed no detectable immunostaining for NQO1 either in the cytosol or on mitotic spindles (Fig 2). These data confirm that NQO1 is the target of monoclonal antibody clone A180 and that the immunostaining of mitotic spindles by anti-NQO1 antibodies from clone A180 is not observed when NQO1 protein levels are knockdown.

**Figure 3:**
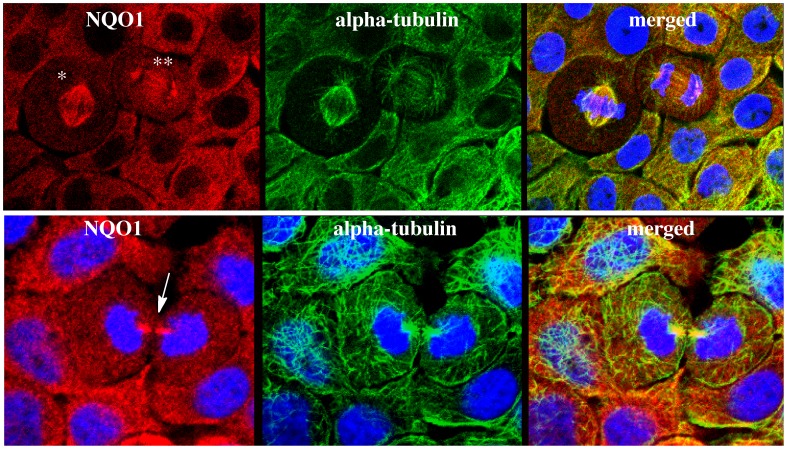
Co-localization of NQO1 and alpha-tubulin in mitotic spindles and midbody region in BxPc-3 human pancreatic cancer cells. Triple immunofluorescent staining for NQO1 (red), alpha-tubulin (green) and nuclei (blue). Association of NQO1 with spindles can be seen in different stages of mitosis including metaphase (*), telophase (**) and the midbody region (arrow) during cytokinesis.

**Figure 4:**
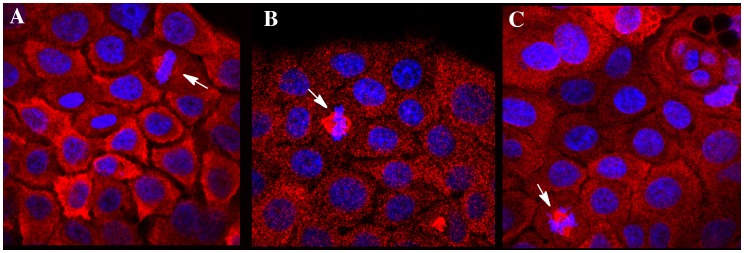
NQO1 inhibitors do not prevent binding of NQO1 to mitotic spindles. Immunostaining for NQO1 (red) in BXPC3 cells pretreated with DMSO (panel A), ES936 (500 nM, panel B) or dicoumarol (50µM, panel C). Cells were pretreated with inhibitors 2 hr before immunostaining. Arrows indicate mitotic cells.

To confirm co-localization of NQO1 with the microtubule containing mitotic spindles, doubling-labeling experiments were performed using antibodies against NQO1 and alpha-tubulin and results from these studies confirmed the co-localization of NQO1 with alpha-tubulin in the mitoic spindles (Fig 3). The association of NQO1 with the mitotic spindles could be observed throughout mitosis from metaphase to cytokinesis (Fig. 3). In cells not undergoing division no obvious co-localization of NQO1 with alpha-tubulin was observed, (see Fig. 3 D–F) suggesting that the association between NQO1 and microtubules is specific for mitotic spindles. Treatment of BxPc-3 cells with the competitive inhibitor dicoumarol or the mechanism-based inhibitor ES936 did not prevent the association of NQO1 with mitotic spindles suggesting that the association of NQO1 with the mitotic spindle is not dependent upon the redox state of NQO1 (Fig. 4).

**Figure 5:**
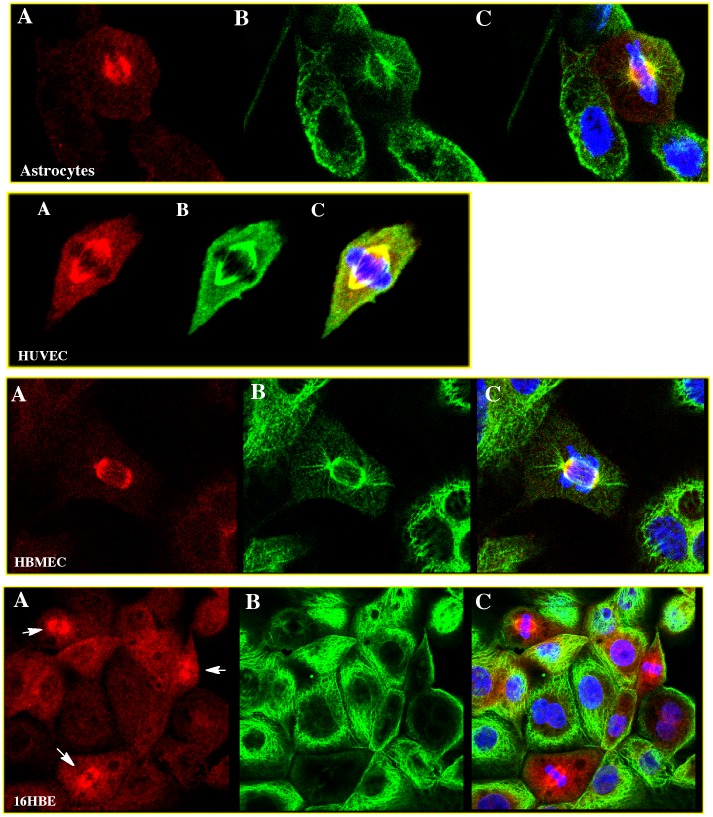
Co-localization of NQO1 and alpha-tubulin with mitotic spindles in human cells. Triple immunofluorescent staining for NQO1 (red, panel A); alpha-tubulin (green, panel B); merged (with DAPI,panel C).

**Figure 6:**
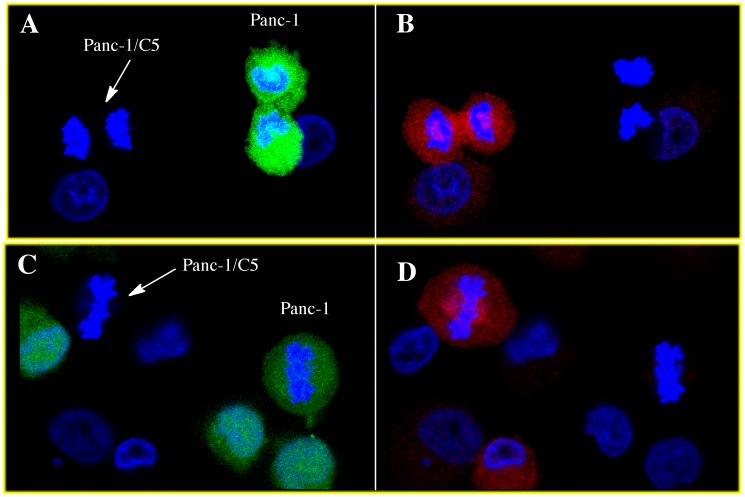
Co-localization of NQO1 with mitotic spindles is not observed in cells homozygous for the NQO1*2 polymorphism. Panc-1 cells which are homozygous for the NQO1*2 polymorphism (NQO1 null), were prelabeled with cell tracker green then mixed with unlabled Panc-1/C5 cells which stably express wild type hNQO1. Following a 24hr coincubation period cells were fixed and immunostained for NQO1 followed by confocal microscopy. Cells were searched until a mitotic Panc-1 cell (green) and a mitotic Panc-1/C5 cell (non-staining) were observed in the same field. Panels A and C, data was collected using 488nm excitation wavelength for cell tracker green fluorescence (green Panc-1 cells). Panels B and D, data was collected using 561nm excitation wavelength (red/NQO1). Co-localization of NQO1 to the mitotic spindles was not observed in NQO1-null Panc-1 cells (green) while co-localization of NQO1 to the mitotic spindle was seen in NQO1 overexpressing Panc-1/C5 cells.

The immunofluorescent staining of NQO1 was performed in many different human cell lines (Fig. 5) including cancer cell lines (pancreatic), immortalized cell lines (HBMEC, 16HBE) and primary cell lines (astrocytes, HUVEC). The human pancreatic cancer cell line Panc-1, which has previously been genotyped as homozygous for the NQO1*2 polymorphism [17] and characterized as NQO1 null, did not demonstrate detectable immunofluorescent staining for NQO1 either in the cytosol or mitotic spindles. Stable expression of NQO1 in the Panc-1 cell line (clone panc-1/C5) resulted in significant staining for NQO1 in the cytosol and mitotic spindles (Fig 6).

**Figure 7:**
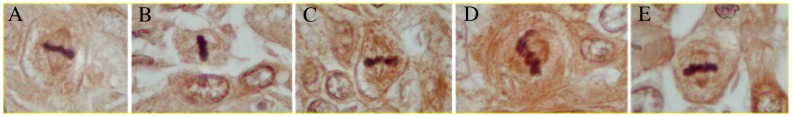
NQO1 localizes to mitotic spindles in human lung cancer cells. Immunohistochemical analysis of NQO1 (DAB, brown) in archival formalin-fixed human squamous lung carcinoma tissue samples. Mitotic figures are visible due to their intense chromatin staining (hemotoxylin).

To determine if NQO1 could be found associated with mitotic spindles in human tissues, we examined human NSCLC tissue samples that had been immunostained for NQO1 in a previous study [18]. Upon examination of squamous cell carcinomas samples mitotic figures were located and immunostaining for NQO1 was clearly visible on the mitotic spindles of these cells (Fig 7). These data support our studies in cell culture that demonstrate the association of NQO1 with mitotic spindles.

## Discussion

These data demonstrate that the quinone oxidoreductase NQO1 associates with the mitotic spindles and suggests that this association with the mitotic spindle is independent of the redox state of NQO1 since immunostaining for NQO1 on spindles was not diminished in the presence of NQO1 inhibitors. While this is the first report of localization of NQO1 with mitotic spindles, previous studies using Xenopus egg extracts indentified NQO1 as a target in a chemical screen of purine-based mitotic disrupters [19]. In this study, diminutol (2-(1R-isopropyl-2-hydroxyethylamino)-6-(3-aminophenylthio)-9-isopropylpurine), which prevents microtubule polymerization, was also shown to inhibit NQO1 catalytic activity via competitive binding with NAD(P)H. These same studies demonstrated immuno-depletion of NQO1 from *Xenopus* egg extracts resulted in defects in spindle assembly [19]. Despite these findings that suggested NQO1 catalytic activity may be linked to spindle assembly in Xenopus eggs, the authors did not demonstrate localization of NQO1 with spindles [19]. Unlike studies in Xenopus eggs, our studies in human cells have shown that NQO1 can be found associated with the mitotic spindles; however, treatment of human cells with the NQO1 mechanism-based inhibitor ES936, which resulted in >95% inhibition of NQO1 catalytic activity, did not alter the growth rate of these cells [20]. These data suggest that in human cells despite the binding of NQO1 to the mitotic spindles NQO1 most likely does not play a direct role in regulating mitosis. These observations are reinforced by studies in human cell lines that lack NQO1 activity and protein due to homozygous expression of the NQO1*2 polymorphism. Many commonly used human cells lines (BE, MDA231, MDA468, Panc-1, Caco-2) lack NQO1 activity and protein due to homozygous expression of the NQO1*2 polymorphism and for many of these cell lines isogenic pairs have been created following the stable transfection of wild type NQO1. The reintroduction of wild type NQO1 back into these cell lines did not significantly alter the growth rate when compared to their NQO1 null isogenic parental cells [21]. Taken together, these data imply that in human cells NQO1 does not play a major role in regulating mitosis but instead NQO1 may act in a protective role.

The ability of NQO1 to associate with the mitotic spindles in human cells while still maintaining catalytic activity has implications for both quinone detoxification and activation reactions in close proximity to critical mitotic apparatus. The reduction of quinones by NQO1 could result in protection of vulnerable microtubules from attack by electrophilic quinones. The benzene metabolite *p*-benzoquinone has been shown in cell free systems to react readily with sulfhydryl groups on microtubule proteins and inhibit assembly into microtubules [22]. Alternatively, NQO1 could be the source of high levels of reactive oxygen species due to redox cycling of unstable hydroquinones formed in close proximity to the mitotic apparatus.

An alternative catalytic function of NQO1 may be to aid in DNA repair during mitosis through the generation of NAD^+^ an essential cofactor for proteins including the family of poly (ADP ribose) polymerase (PARP) enzymes and the sirtuins. PARP enzymes have been shown to play an important role in the repair of single strand DNA breaks [23] while SIRT6 has been proposed to aid in DNA repair under conditions of oxidative stress by binding directly to PARP1 and stimulating PARP1 poly-ADP-ribosylase activity [24].

From our studies it is unclear whether NQO1 binds directly to microtubules in mitotic spindles or binds to a protein complex that associates with the mitotic spindles. NQO1 has many characteristics of a microtubule binding protein including a net positive charge and multiple domains containing clusters of positively charged amino acids [25], [26], [27]. Alternatively, NQO1 could be binding to another protein or protein complex that is tethered to the mitotic spindle. In addition to its major role in quinone metabolism NQO1 has emerged as a regulator of 20 S proteasomal degradation [28]. A growing number of proteins have been shown to be protected from ubiquitin-independent 20 S proteasome degradation by NQO1 including p53, p63, p73 and ornithine decarboxylase [14], [29], [30], [31]. Interestingly, immunofluorescence studies have previously demonstrated co-localization of p53 with centrosomes and mitotic spindles in human cell lines [32] and co-localization of the 20 S proteasome to centrosomes and mitotic spindles in rat Schwann cells [33]. Whether NQO1 interacts with these proteins on the spindle to facilitate in the stabilization and redistribution of selected proteins such as p53 during mitosis is currently under investigation. Recently, it has been shown that persistent expression of NQO1 via p62-mediated Nrf2 activation facilitated p53-dependent mitotic catastrophe supporting a role for NQO1 in the stabilization of p53 during mitosis [34].

In summary, these data report the association of NQO1 with mitotic spindles in human cells using immunofluorescent staining and suggest that the association of NQO1 with spindles may involve protection against electrophic quinones, aid in the generation of oxidized pyridine nucleotides or shield selected proteins from 20 S degradation.

## Materials and Methods

### Reagents

Anti-NQO1 mouse monoclonal antibody (IgG_1_, clone A180) was developed in our laboratory against full-length recombinant human NQO1 protein and hybridoma supernatant (RPMI1640 containing 10% fetal bovine serum) from clone A180 was used for these studies (dilution 1∶5–1∶20). Affinity purified anti-NQO1 monoclonal antibody from clone A180 was obtained commercially from Cell Signaling Technologies (Danvers, MA) and used at a dilution of 1∶200. Anti-alpha tubulin rabbit polyclonal antibody was purchased from Abcam, (Cambridge MA, Cat#15246, dilution 1∶10,000). DyLight 488 and DyLight 544 conjugated goat anti-mouse IgG and DyLight 488 conjugated goat anti-rabbit IgG antibodies were purchased from Jackson ImmunoResearch Laboratories (West Grove PA). 4′,6-diamidino-2-phenylindole (DAPI,) dicoumarol and doxycycline were obtained from Sigma (St. Louis MO). The NQO1 mechanism-based inhibitor ES936 (5-methoxy-1,2-dimethyl-3-[(4-nitrophenoxy)methyl]indole-4,7-dione) was synthesized in the laboratory of Dr. Christopher J. Moody, School of Chemistry, University of Nottingham, U.K [35].

### Cell Lines and Tissues

BXPC3 and Panc-1 human pancreatic cancer cell lines were obtained from ATCC (Manassas, VA) and grown in RPMI 1640 containing 5% (v/v) fetal bovine serum and 1% (v/v) penicillin/streptomycin. Panc-1 cells have previously been genotyped as homozygous for the NQO1*2 polymorphism and are NQO1null [17]. Panc-1/C5 cells stably express wildtype NQO1 and were created from parental Panc-1 cells as described [17]. Primary cultures of human umbilical cord vein endothelial cells (HUVEC) were obtained from Cell Applications (San Diego, CA) and grown in Endothelial Cell Growth Media (Cell Applications). Primary human astrocytes were obtained from ScienCell Research Labs (Carlsbad, CA) and grown in Astrocyte Medium (ScienCell Research Labs). Immortalized human bone marrow endothelial cells (HBMEC) were provided by Dr. B.B. Weksler (Cornell University) and grown as previously described [36]. Immortalized human bronchial epithelial cells [37] (16HBE) were provided by Dr. Brian Day (National Jewish Health, Denver CO) and grown in RPMI 1640 containing 5% fetal bovine serum and 1% penicillin/streptomycin. All cells were maintained as monolayers at 37°C in 5% CO_2_.

For immunocytochemical studies all cells were plated onto glass coverslips (18 mm×0.15 mm circle, Fisher Scientific) in 100 mm tissue cultures dishes 3 days prior to analysis. For studies using NQO1 inhibitors ES936 and dicoumarol cells were incubated in fresh media (2 ml) containing inhibitors (500 nM ES936 or 50 µM dicoumarol) for 2 hr. The generation of a doxycycline-inducible NQO1 knock-down cell line was performed using a TRIPZ inducible shRNA plasmid for human NQO1 (Open Biosystems, Lafayette, CO). To create a stable knockdown cell line, 2×10^6^ BxPc3 cells were electroporated with 20 ug of TRIPZ inducible shRNA plasmid for human NQO1 and transfected cells were selected in normal culture media containing 3 µg/ml puromycin. The antibiotic resistant cells were induced with 300 ng/ml doxycyclin for 48 h and then cells were sorted for turbo red fluorescence (553excitation/574emission) using the Legacy MoFlo sorter (University of Colorado Cancer Center). Sorted cells were maintained in complete media containing 500 ng/ml doxycycline and knockdown of NQO1 was confirmed by immunoblot analysis.

Coincubation studies with Panc-1 cells and Panc-1/C5 cells were performed using the following method. Panc-1 cells (1×10^6^) were treated with 5 µM Cell Tracker Green (Invitrogen, Carlsbad, CA) in serum-free media in suspension at 37°C. After 1 hr Panc-1 cells were collected by centrifugation, washed in PBS and then mixed with unlabeled Panc-1/C5 cells (1×10^6^) and plated onto glass coverslips in complete media. After 24 hr the coverslips were fixed for immunocytochemisty as described below.

Achieved formalin fixed human squamous lung cancer tissue was obtained from the University of Colorado Cancer Center and immunostained for NQO1 as previously described [18].

### Immunocytochemistry

Immunocytochemistry was performed on cell lines grown on glass coverslips using the following methods. All steps were performed at room temperature. Cells were washed in phosphate buffered saline (PBS) then fixed in 3.7% (v/v) formaldehyde in PBS for 12 min, rinsed with PBS, then permeabilize with 0.15% (v/v) Triton-X100 in PBS for 10 min. Cells were then blocked in RPMI1640 containing 5% fetal bovine serum for 1 hr at room temperature. Coverslips were then transferred to a 6-well plate (single coverslip/well) and primary antibodies diluted in TBST (10 mM Tris-HCl, pH 8 containing 150 mM NaCl and 0.4% (v/v) Tween-20), were added (2 ml/well). After 90 min the primary antibodies were removed, the cells were gently rinsed with TBST, and secondary antibodies (diluted in TBST) were then added (2 ml/well) for 30 min. DAPI (1 µg/ml) was included with the secondary antibody. After 30 min coverslips were rinsed in TBST, briefly immersed in deionized/distilled H_2_0, inverted, and mounted on to glass microscope slides using SuperMount (Biogenex, San Ramon, CA). Stained cells were examined using a Nikon TE2000E2 inverted microscope equipped with a C1-Plus confocal system (lasers 402 nm, 488 nm, 514 nm, Nikon Instruments, Melville, NY).

## Supporting Information

Figure S1 **No fluorescent immunostaining of mitotic spindles in the absence of anti-NQO1 primary antibody.** When unconditioned RPMI1640 containing 10% fetal bovine serum was substituted for conditioned hybridoma clone A180 supernatant no immunostaining of cells or mitotic spindles could be observed. Panel A, acquired with confocal settings used for Figure 1; Panel B, acquired with confocal settings greatly enhanced for detection of Dylight 549 nm (red). Arrows indicate mitotic cells.
(TIF)

Figure S2 **NQO1 immunostaining in BxPc-3 cells using hybridoma supernatant or purified antibody.** Identical immunostaining of NQO1 in BxPc-3 cells was observed using either (A) hybridoma tissue culture media from clone A180 diluted 1∶10 or (B) affinity purified mouse IgG from clone A180 (Cell Signaling Technologies) diluted 1∶200.
(TIF)
